# Giant peak of the Inverse Faraday effect in the band gap of magnetophotonic microcavity

**DOI:** 10.1038/s41598-018-29294-w

**Published:** 2018-07-30

**Authors:** Mikhail A. Kozhaev, Alexander I. Chernov, Daria A. Sylgacheva, Alexander N. Shaposhnikov, Anatoly R. Prokopov, Vladimir N. Berzhansky, Anatoly K. Zvezdin, Vladimir I. Belotelov

**Affiliations:** 1 Russian Quantum Center, 45 Skolkovskoye shosse, Moscow, 121353 Russia; 2 Prokhorov General Physics Institute of the Russian Academy of Science, 38 Vavilov Street, Moscow, 119991 Russia; 3 Faculty of Physics, Lomonosov Moscow State University, Leninskie Gory, Moscow, 119991 Russia; 4 Vernadsky Crimean Federal University, 4 Vernadskogo Prospekt, Simferopol, 295007 Russia; 5 P.N. Lebedev Physical Institute of the Russian Academy of Sciences, 53 Leninskiy Prospekt, Moscow, 119991 Russia

## Abstract

Optical impact on the spin system in a magnetically ordered medium provides a unique possibility for local manipulation of magnetization at subpicosecond time scales. One of the mechanisms of the optical manipulation is related to the inverse Faraday effect (IFE). Usually the IFE is observed in crystals and magnetic films on a substrate. Here we demonstrate the IFE induced by fs-laser pulses in the magnetic film inside the magnetophotonic microcavity. Spectral dependence of the IFE on the laser pulse wavelength in the band gap of the magnetophotonic microcavity has a sharp peak leading to a significant enhancement of the IFE. This phenomenon is explained by strong confinement of the electromagnetic energy within the magnetic film. Calculated near field distribution of the IFE effective magnetic field indicates its subwavelength localization within 30 nm along the film thickness. These excited volumes can be shifted along the sample depth via e.g. changing frequency of the laser pulses. The obtained results open a way for ultrafast optical control of magnetization at subwavelength scales.

## Introduction

Optical control of the magnetization at ultrashort time scales is of prime interest^1–6^ in context of the data processing and spintronic applications^7,8^. The inverse Faraday effect (IFE) provides a new way for efficient control of spins at GHz and THz rates^1^. Circularly polarized laser pulses can induce the IFE effective magnetic field of about 10^2^–10^4^ Oe in a magnetic dielectric and excite the magnetization precession leading to magnetostatic spin waves^9–18^. By now, the IFE has been investigated only in a freestanding crystals and films on a substrate. In this case optical pulses influence on the magnetization almost uniformly along the whole depth of the magnetic film. In the spectral range where dispersion of the optical and magneto-optical properties of a magnetic sample is small the IFE depends on the laser pulse wavelength marginally.

At the same time, light localization within a magnetic layer makes the light-matter interaction more efficient and provides notable resonances in optical spectra resulting in a significant enhancement of different magneto-optical effects^19–27^. For the magnetic structures containing conductive media the key role is played by surface plasmon-polaritons^19–24^. The other type of localization is achieved in all-dielectric magnetic structures: magnetophotonic crystals and microcavities. The magnetophotonic crystals contain a periodic magnetic cell, while the magnetophotonic microcavities (MPMC) consist of a subwavelength-size magnetic medium surrounded by a photonic crystal^25–27^. In the latter case the light localization arises at the microcavity resonance spectrally located inside the photonic band gap^26,27^.

Optical control of magnetization using magnetic nanostructures has been demonstrated in GdFeCo and TbFeCo films covered with gold double-wire antennas and rectangular apertures in a gold film^28,29^. Subwavelength localization of the magnetic area influenced by light was achieved. Recent paper^30^ describes the plasmon-induced demagnetization in nickel nanoparticles. The IFE in the samples covered by the nano-size apertures was theoretically investigated in the context of possible application for high-density all-optical magnetic recording^31^.

Due to the large optical losses in metals the plasmonic resonances are quite broad, that does not allow obtaining large values of the IFE and rather leads to thermal effects. In this respect, all-dielectric structures are more advantageous since they provide optical resonances with high optical quality factors^25,26^. The IFE in all-dielectric structures has not yet been experimentally considered.

In this work, we investigate the influence of the optical confinement on the IFE in the all-dielectric MPMCs. We excite the magnetization dynamics by the fs-laser pulses in the MPMC to demonstrate that the IFE becomes very sensitive to the excitation laser wavelength and increases significantly at the microcavity resonance in the photonic band gap.

## Results

**Figure 1:**
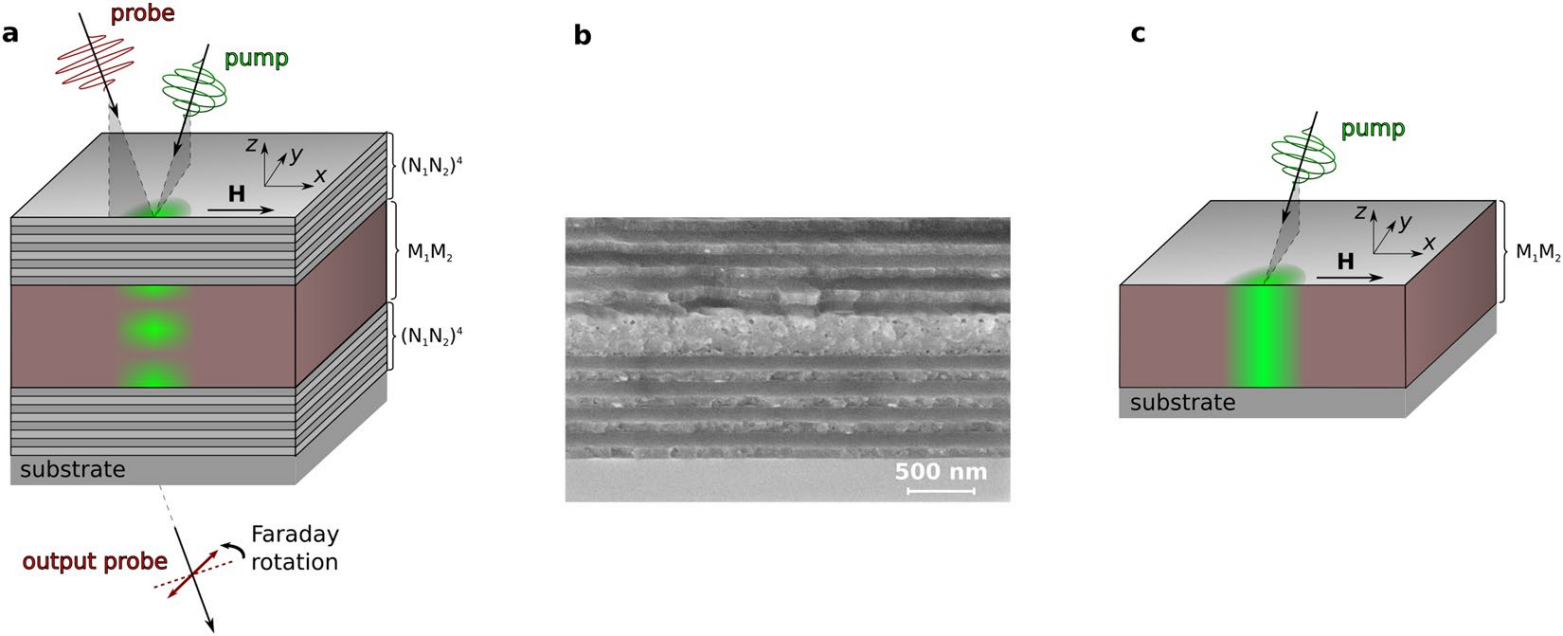
The 3D Inverse Faraday effect in a magnetophotonic microcavity. (**a**) Scheme of the experiment. The sample is a magnetophotonic microcavity formed by the magnetic film (brown) sandwiched in between two nonmagnetic Bragg mirrors formed by several pairs of the dielectric layers N1 and N2 (gray). The circularly polarized pump excites the magnetic film and the linearly polarized probe is used to observe the magnetization dynamics at some time delay. (**b**) SEM image of the MPMC sample cross-section. (**a**,**c**) Calculated distributions of the optically generated effective magnetic field inside the magnetic layer of the MPMC (**a**) and inside a single magnetic film (**c**) are shown on the front side of the samples by green color demonstrating the 3D and 2D localization of the IFE, respectively.

**Figure 2:**
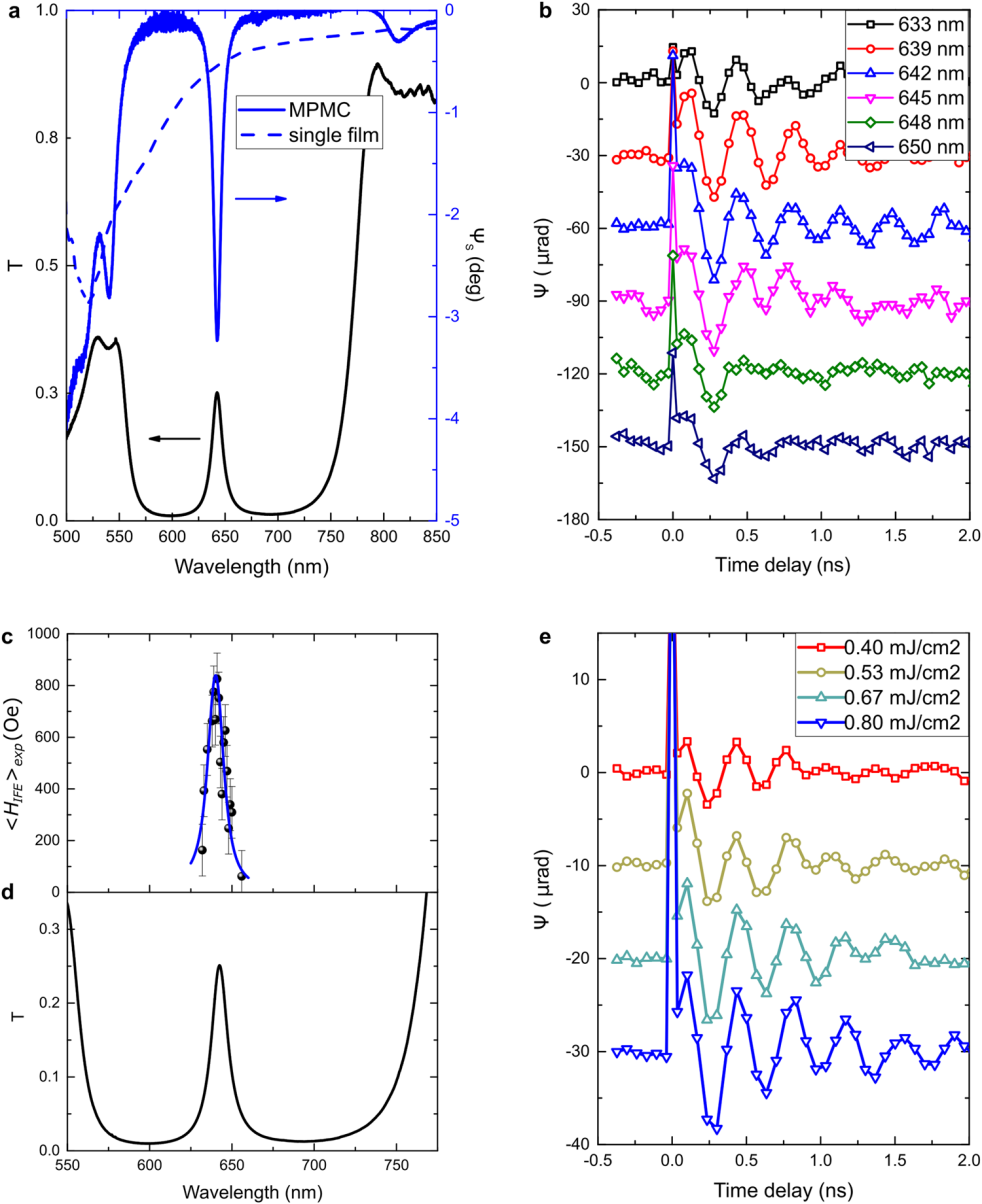
Laser pulse excited magnetization dynamics in MPMC. (**a**) Spectra of the optical transmittance (black curve) and Faraday rotation (blue curve) for the MPMC and the single magnetic film similar to the microcavity one (dashed blue curve) fully magnetized out-of-plane. (**b**) Time-resolved change of the Faraday rotation indicating the magnetization precession at different excitation wavelengths around the cavity resonance within the photonic band gap. All curves have offsets for clarity of representation. The pump fluence is 0.66 mJ/cm^2^. (**c**) Averaged over the thickness of the magnetic film the normal component of the IFE magnetic field versus the pump wavelength: found from the experimental data, 〈*H*_*IFE*_〉_*exp*_ (black spheres), and calculated from the electromagnetic field distribution, 〈*H*_*IFE*_〉_*calc*_ (solid blue curve). (**d**) Optical transmittance of the pump beam versus the pump wavelength. Angle of light incidence is 43°. (**e**) Time-resolved change of the Faraday rotation indicating the magnetization precession excited by the pump beam at λ = 640 nm (near the MPMC resonance) for different values of fluence from 0.40 to 0.80 mJ/cm^2^. All curves have offsets for clarity of representation. External magnetic field is 890 Oe.

For performing the experiments, we used the MPMC with a bismuth iron-garnet magnetic film sandwiched between two nonmagnetic dielectric Bragg mirrors (Fig. 1a). Iron-garnet films doped with bismuth provide a large Faraday effect and have a relatively low optical absorption in the long-wavelengths visible spectral range^32,33^. Doping allows achieving a high magneto-optical figure of merit in the MPMC. The magnetic film has an out-of-plane anisotropy and consists of the auxiliary layer (M1) and the main layer (M2). The M1-layer is needed to deposit the high quality M2-layer with large Bi concentration. For the 43° light incidence the cavity mode is excited in the photonic band gap center at λ_0_ = 642 nm (Fig. 2a). It is accompanied by the 5-fold enhancement of the Faraday effect with respect to the single magnetic film. The optically excited spin dynamics in the MPMC was investigated by the pump-probe experimental technique. The circularly polarized 150 fs pump-pulse was used to excite the magnetization precession due to the IFE: an effective magnetic field, H_IFE_, appears in the sample during the pulse propagation.

The observed oscillations of the Faraday angle of the probe beam, *Ψ*, can be described by the harmonic function with decaying amplitude ${\Psi }(t)={{\Psi }}_{m}\exp (\,-t/\tau )\sin \,\omega t$, where ***Ψ***_***m***_ is an initial amplitude of *Ψ*, *τ* is a decay time of the precession, and *ω* is a precession frequency (Fig. 2b). Amplitude of the observed oscillations strongly depends on the pump wavelength that is varied near the cavity resonance. Full-width at half-maximum (FWHM) of the ***Ψ***_***m***_(***λ***) resonance is Δ*λ* = 12 nm, that is equal to the FWHM of the transmission peak.

It should be noted that the decay time is also sensitive to the pump wavelength: the longest decay appears at the resonance wavelength. In order to clarify this point we changed fluence of the pump beam adjusted to the MPMC resonance (Fig. 2e). It can be seen that the decay time gets larger with an increase of the fluence. Therefore, higher amplitude of the optical field inside the magnetic film with Gd-ions leads to longer magnetization precession. It allows us to suppose that the observed increase of the decay time by approaching wavelength of the microcavity resonance might be due to the optical power concentration in the magnetic layer.

As duration of the laser pulses, Δ*t*, is much smaller than the ferromagnetic resonance period, the magnetization dynamics can be considered as the decaying precession around the external magnetic field with the initial conditions determined by H_*IFE*_. If a direction of the magnetization vector is described by the angle *θ* between the magnetization and the film plane, then $\theta (t)={\theta }_{m}{e}^{-\frac{t}{\tau }}\,\sin \,\omega t$, where *θ*_*m*_ is an initial amplitude of *θ*. The precession amplitude is mainly determined by the normal component of the IFE magnetic field, *H*_*IFE*_:

1 $${\theta }_{m}=\gamma {(1+{H}_{a}/H)}^{-1/2}{H}_{IFE}{\rm{\Delta }}t,$$

where *γ* is a gyromagnetic ratio, ${H}_{a}=4\pi M-2{K}_{U}/M$, *M* is a saturation magnetization and *K*_*U*_ is an uniaxial anisotropy constant^34^ (Supplementary material). Therefore, *H*_*IFE*_ can be found from measuring the precession amplitude.

In the magnetic film inside the MPMC the optical field distribution over the film thickness is not uniform, that causes some distribution of *H*_*IFE*_(*z*). In accordance to Eq. (1) ${\theta }_{m}(z)\sim {H}_{IFE}(z)$. With time the distribution of *θ*(*z*) becomes smoother since spins in the adjacent areas of the film interact by the dipole-dipole and exchange interactions.

Depth dependent magnetization precession *θ*(*z*) is detected by the probe pulse. As the probe pulse also propagates through the MPMC the distribution of its electric field, **E**_*pr*_(*z*), in the magnetic film is not uniform as well. Consequently, the observed signal accounts a relative distribution of *θ*_*m*_(*z*) and E_*pr*_(*z*). However, in order to estimate the out-of-plane component of the IFE magnetic field, 〈***H***_***IFE***_〉_***exp***_, from the experiment, one can assume that the probe beam measures averaged over the film thickness precession angle 〈*θ*〉 and ${\Psi }_{m}={\Psi }_{s}\,\cos \,\beta \langle {\theta }_{m}\rangle$, where Ψ_*s*_ is the Faraday rotation of the film magnetically saturated out-of-plane and *β* is the refraction angle of the incident light inside the magnetic film. Here cos *β* takes into account that the Faraday angle is proportional to the projection of magnetization on the light wavevector.

Calculations confirm that this assumption leads to rather small inaccuracy, but allows a notable simplification of the formulas. Consequently, average value of the IFE magnetic field, 〈*H*_*IFE*_〉_*exp*_, can be found from the experimental data as:

2 $${\langle {H}_{IFE}\rangle }_{exp}=\frac{\sqrt{1+{H}_{a}/H}}{\gamma \,\cos \,\beta {\rm{\Delta }}t}(\frac{{{\Psi }}_{m}}{{{\Psi }}_{s}})\cdot $$

The value of the IFE strongly depends on the pump wavelength (black spheres in Fig. 2c) demonstrating a giant peak in the photonic band gap (*λ* = 642 nm) (compare Fig. 2c,d). At the resonance wavelength 〈*H*_*IFE*_〉_*exp*_ reaches 840 Oe.

**Figure 3:**
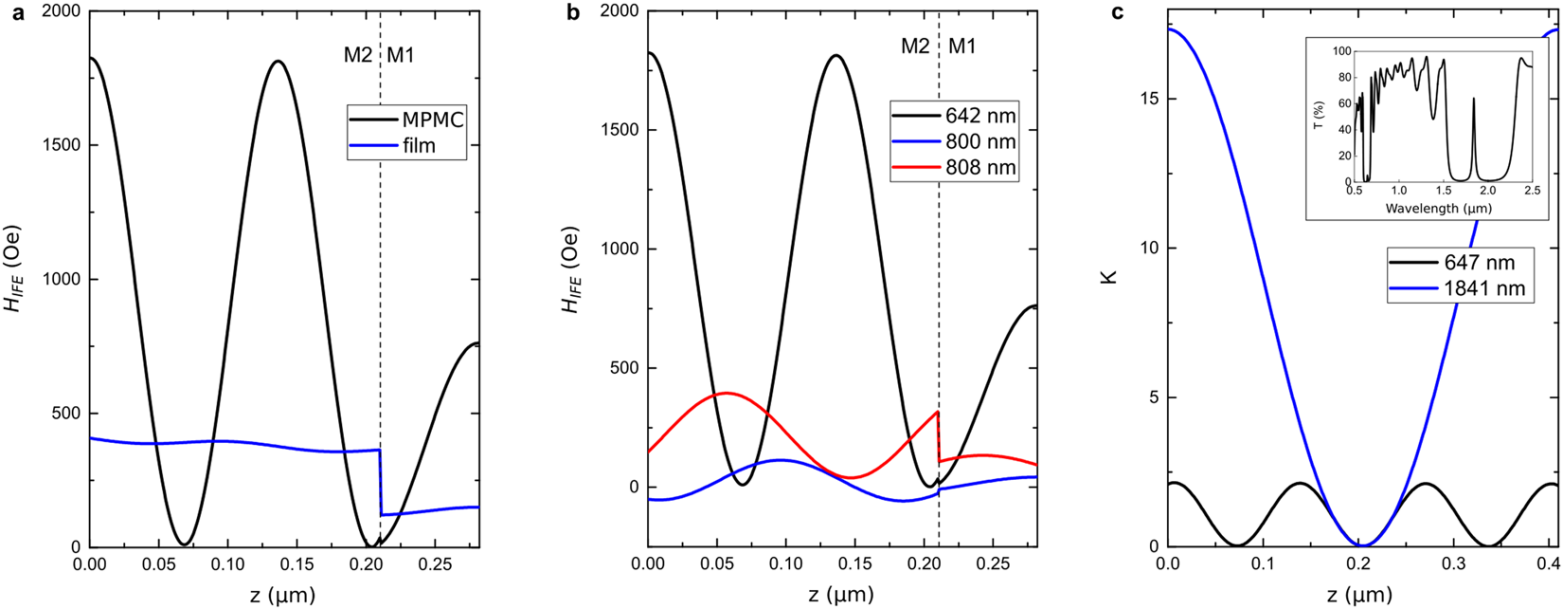
Calculated distribution of the IFE effective magnetic field in the experimentally studied MPMC (**a**,**b**) and in the MPMC with three times thicker layers (**c**). (**a**) The IFE magnetic field in the magnetic film of the experimentally studied MPMC at the cavity resonance (black curve) and in the same single magnetic film on the substrate (blue curve) at *λ* = 642 nm. (**b**) The IFE magnetic field in the magnetic film of the experimentally studied MPMC at *λ* = 642 nm (black curve), 800 nm (blue curve), and 808 nm (red curve). In (**a**) and (**b**) two magnetic layers are shown: the main one (M1, 0 < *z* < 210 nm) and the auxiliary one (M2, 210 < *z* < 282 nm). (**c**) The IFE magnetic field enhancement factor, *K*, in the MPMC with the main resonance at *λ* = 1841 nm as compared to the single magnetic film at the 1-st order (*λ* = 1841 nm, blue curve) and 2-nd order (*λ* = 647 nm, black curve) cavity resonances. The MPMC in (**c**) has Bragg mirrors of four pairs of 228-nm-thick TiO_2_ and 351-nm-thick SiO_2_ layers, and the magnetic film of 410-nm-thick Bi_1.5_Gd_1.5_Fe_4.5_Al_0.5_O_12_. Area of the magnetic layer is shown (0 < *z* < 410 nm). Inset: transmission spectrum of the MPMC with the main resonance at *λ* = 1841 nm.

On the other hand, the IFE magnetic field is determined by the distribution of the pump electric field **E** inside the magnetic film^1^:

3 $${{\bf{H}}}_{IFE}=-\,\frac{{\varepsilon }_{0}}{{\mu }_{0}}\frac{g}{M}{\rm{Im}}([{\bf{E}}\times {{\bf{E}}}^{\ast }]),$$

where *ε*_0_ and *μ*_0_ are free space permittivity and permeability, respectively, and *g* is a medium gyration. To calculate the distribution of **E**(*z*) we used the transfer matrix method^35^ (Supplementary material). At *λ* = 642 nm Eq. (3) gives *H*_*IFE*_(z) with the maximum value of (*H*_*IFE*_)_*calc*_ = 1825 Oe and the average of 〈*H*_*IFE*_〉_*calc*_ = 850 Oe (Fig. 3a, black curve). The latter is in nice agreement with 〈*H*_*IFE*_〉_*exp*_ found from the experimental data via Eq. (2). Calculations for other pump wavelengths around the cavity resonance also follow the experimental results with good precision (solid blue curve in Fig. 2c). It confirms applicability of Eqs (1–3) in our case.

At the cavity resonance (*λ* = 642 nm) the function of *H*_*IFE*_(*z*) in each magnetic layer takes harmonic form: ${H}_{IFE}(z)={H}_{IFEm}{\cos }^{2}\frac{2\pi \sqrt{\varepsilon }}{{\lambda }_{0}}z$, where *ε* is the magnetic layer permittivity (black curve in Fig. 3a). The value of *H*_*IFEm*_ exceeds by about 5 times the IFE field in the same single magnetic film on a substrate, *H*_*IFE*0_ (blue curve in Fig. 3a). Distribution of *H*_*IFE*0_(*z*) is close to uniform. The discontinuities of *H*_*IFE*_(*z*) are due to different values of gyration of two sub-layers constituting the magnetic film.

The field *H*_*IFE*_(*z*) has three maxima within the magnetic film at *z* = *ζ*_*i*_. Two of them are at the interfaces with the Bragg mirrors: *ζ*_*i*_ = 0 with FWHM of $\frac{{\lambda }_{0}}{8\sqrt{{\varepsilon }_{1}}}=33\,{\rm{nm}}$ and *ζ*_3_ = 282 nm with FWHM of $\frac{{\lambda }_{0}}{8\sqrt{{\varepsilon }_{2}}}=37\,{\rm{nm}}$. The intermediate maximum is at ${\zeta }_{2}=\frac{{\lambda }_{0}}{2\sqrt{{\varepsilon }_{1}}}=130\,{\rm{nm}}$ and has FWHM of $\frac{{\lambda }_{0}}{4\sqrt{{\varepsilon }_{1}}}=65\,{\rm{nm}}$. Therefore, laser pulses excite the spin dynamics in the magnetic film locally in depth. Moreover, focusing of the pulses also adds the lateral locality. As a result, in the MPMC we deal with the three-dimensional localization of the IFE in the cylindrical regions, which are 9 µm in diameter and only several tens of nanometers in height.

Furthermore, position of the IFE localization regions inside the magnetic film is tunable by variation of the pump wavelength or incidence angle. In particular, the IFE-regions are shifted by about 60 nm in depth, if the pump wavelength is changed from *λ* = 642 nm to *λ* = 808 nm, corresponding to the edge of the photonic band gap (Fig. 3b). Therefore, spins in the film layers that are not directly excited at *λ* = 642 nm, become accessible at *λ* = 808 nm, and vice versa. At some wavelength range (around *λ* = 800 nm) the direction of the IFE magnetic field changes by the opposite one within only 100 nm depth (blue curve in Fig. 3b). Opposite direction of *H*_*IFE*_ is due to the oblique incidence of the pump pulse at the wavelengths around the edge of the photonic band gap. Under these conditions phase difference between *E*_*x*_ and *E*_*y*_ optical field components leads to the opposite sign of ${\rm{Im}}([{\bf{E}}\times {{\bf{E}}}^{\ast }])$ describing the IFE (see Eq. (3)).

If the MPMC parameters are chosen in order to obtain the cavity modes of several orders in the transparency wavelength range of the magnetic film, then the IFE localization regions can be drastically changed by switching the pump between the cavity resonances (Fig. 3c). The difference in the enhancement coefficient $K=\frac{{H}_{IFEm}}{{H}_{IFE0}}$ at *λ* = 647 nm and *λ* = 1841 nm is due to much higher absorption of iron-garnets in the visible range. The maximum localization length is $\frac{{\lambda }_{0}}{8\sqrt{{\rm{\varepsilon }}}}$ and, therefore, it becomes smaller for the higher-order resonances. However, its further decrease is limited by the absorption at short-wavelengths. For iron-garnets the acceptable absorption range starts at λ > 600 nm, that implies best localization of 35 nm.

Variation of the probe wavelength would also give additional advantage as it modifies distribution of the probing electromagnetic field and therefore provides sensitivity to the magnetization dynamics at different parts inside the magnetic film.

Resonance increase and localization of the IFE in the small regions inside the magnetic film at the wavelength of the cavity mode points out that the optical confinement is crucial for these phenomena. It can also be considered in terms of the increase of the optical density states in the cavity with respect to the free space in accordance to the Purcell effect.

The optical confinement is characterized by the quality factor, *Q*, defined by the ratio of the cavity resonance wavelength, *λ*_0_, to the resonance full-width at half maximum, Δ*λ*: $Q={\lambda }_{0}/{\rm{\Delta }}\lambda$. For the definite materials of the MPMC *Q* is determined by the number of layers in the Bragg mirrors.

**Figure 4:**
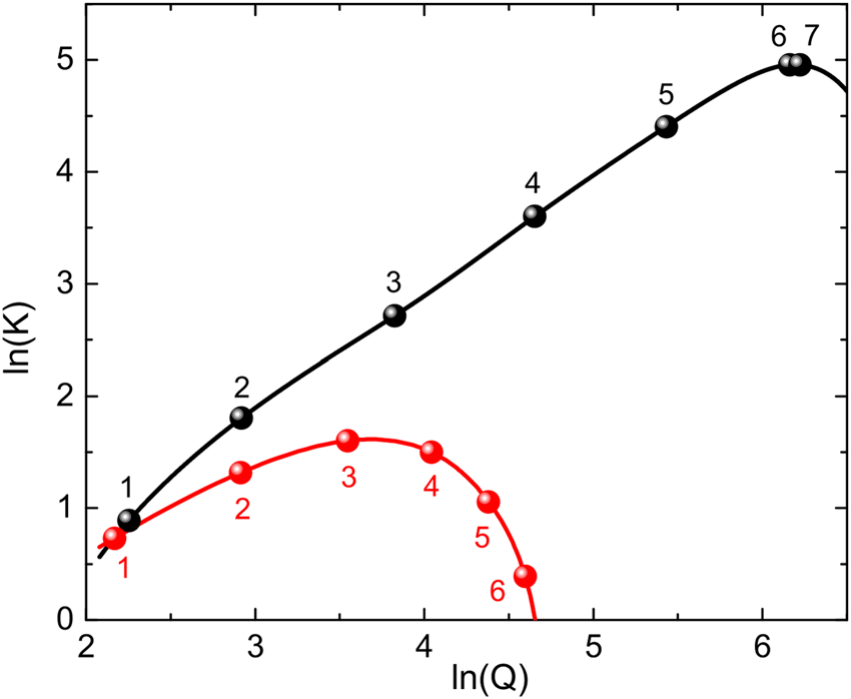
Enhancement factor of the inverse Faraday effect versus quality factor of the magnetophotonic microcavities. Two cases of different optical losses in the iron-garnet layers are considered: at *λ*_0_ = 642 nm (red curve) and at *λ*_0_ = 1400 nm (black curve). Numbers near circles indicate the number of the N_1_N_2_ dielectric layer pairs in the Bragg mirrors of the MPMC.

For the experimentally studied MPMC the absorption of the magnetic film makes the dependence of the enhancement coefficient *K*(*Q*) non-monotonic (Fig. 4). At the level of absorption of the experimentally studied magnetic layers the optimal *Q* corresponds to three pairs of TiO_2_/SiO_2_ layers in the MPMC Bragg mirrors. Consequently, the investigated MPMC sample is close to the optimal one. However, at the resonance wavelength of *λ*_0 _= 1400 nm the absorption of the magnetic film is more than 550 times smaller which leads to the linear dependence of *K*(*Q*) for up to the 6 pairs of the TiO_2_/SiO_2_ layers (*Q* = 470) and promises maximum magnitude of *K* = 110 (Fig. 4, black curve).

## Discussion

We have demonstrated that magnetophotonic microcavities provide a giant peak in the dependence of the inverse Faraday effect on the pump wavelength and a significant enhancement of its value. In particular, the experimentally studied MPMC gives a 5-time increase of the IFE with respect to a single film.

The enhancement factor is related to the quality factor of the microcavity and for this reason it is more advantageous to use dielectric Bragg mirrors rather than the metallic ones and operate in the transparency wavelength range of the magnetic film.

Moreover, calculation of the optical field distribution in the MPMC reveals a subwavelength localization of the IFE along the film thickness down to tens of nanometers. Distribution of the IFE magnetic field in the magnetic film can be varied with the pump wavelength or the incidence angle of the fs-laser pulses. This feature can be used to access specific spins in the magnetophotonic microcavity. Here we have found a shift of the IFE-region by about 60 nm for the pump wavelength change from 642 nm to 808 nm.

Optical confinement might allow excitation of spin wave resonances in the magnetic system, i.e. magnetic modes, which are characterized by non-uniform distribution over the film thickness^36^. Further development of our concept of the IFE in structures with optical confinement can be performed by the implementation of different types of plasmonic or all-dielectric nanoantennas to concentrate the optical fields and the inverse Faraday effect within even smaller volumes with a thickness of about ten nanometers.

## Methods

### Magnetophotonic microcavity sample

The MPMC structure consists of the magnetic film and two non-magnetic dielectric Bragg mirrors. The iron-garnet magnetic film was deposited in the argon-oxygen mixture by reactive ion beam sputtering of two targets with different compositions^37^. It includes two layers M1 and M2: the auxiliary 72-nm-thick Bi_1.0_Lu_0.5_Gd_1.5_Fe_4.2_Al_0.8_O_12_ layer (the saturation magnetization is $4\pi {M}_{s}=330\,{\rm{G}}{\rm{s}}$, and the uniaxial magnetic anisotropy constant is ${K}_{U}=-0.88\cdot 1{0}^{3}\,{\rm{e}}{\rm{r}}{\rm{g}}/{\rm{c}}{{\rm{m}}}^{3}$) and the main 210-nm-thick Bi_2.5_Gd_0.5_Fe_3.8_Al_1.2_O_12_ layer ($4\pi {M}_{s}=360\,{\rm{G}}{\rm{s}}$ and ${K}_{U}=-1.05\cdot 1{0}^{3}\,{\rm{e}}{\rm{r}}{\rm{g}}/{\rm{c}}{{\rm{m}}}^{3}$), respectively. The auxiliary layer is needed to deposit high quality main layer with high Bi content and large specific Faraday effect. Each Bragg mirror of the MPMC consists of four pairs of alternating 76-nm-thick TiO_2_ (N1) and 117-nm-thick SiO_2_ (N2) layers grown on the fused quartz substrate. The structure is well represented in the SEM image obtained with FESEM (ZEISS Supra 40).

### Pump-probe measurements

To investigate the magnetization precession excitation by laser pulses we used the pump-probe experimental technique. The laser system (Newport Mai Tai HP Ti:Sapphire laser and Spectra-Physics Inspire Auto 100 optical parametric oscillator) emits at 80.54 MHz repetition rate pairs of 150-fs-pulses: the pulse at a wavelength tunable from *λ* = 500 nm to 680 nm and the pulse at λ = 820 nm. The first one (the pump pulse) is circularly polarized, has light energy fluence of 0.66 mJ/cm^2^ (calculated for 9 μm diameter) and excites the magnetization precession. The second one (the probe pulse) is 20 times weaker and is used for observation of the magnetization dynamics through the direct Faraday effect, i.e. by measuring variation of the Faraday rotation angle, Ψ, caused by the magnetization precession. The incidence angles of the pump and probe beams are 43° and 30°, respectively. The time delay between the pump and probe pulses is varied from −0.5 to 2.6 ns, where zero time delay corresponds to the simultaneous propagation of the pump and probe pulses through the sample. An external magnetic field, **H**, was applied in-plane to saturate the magnetization.

### Calculation of the electromagnetic field distribution in the MPMC

Distribution of the effective field of the inverse Faraday effect inside the MPMC structures (Fig. 3) was calculated by using the transfer matrix method^35^. Dielectric permittivities and magnetic film gyration were taken from^24^ and found from fitting the experimentally measured transmittance and Faraday rotation spectra of the MPMC with calculated spectra.

At the cavity resonance (*λ* = 642 nm) the magneto-optical parameters of the main and auxiliary layers are *g* = 0.0182 and *g* = 0.0061, respectively, while the absorption coefficients are *α*_1_ = 2600 cm^−1^ and *α*_2_ = 1990 cm^−1^, respectively. At the wavelength of *λ* = 1400 nm *α*_1_ = 4.9 cm^−1^ and *α*_2_ = 4.0 cm^−1^, and at *λ* = 1841 nm *α*_1_ = 3.9 cm^−1^ and *α*_2_ = 3.2 cm^−1^.

## Electronic supplementary material

SUPPLEMENTARY INFORMATION
