# Supplementary material for: Giant peak of the Inverse Faraday effect in the band gap of magnetophotonic microcavity
Source: Sci Rep. 2018 Jul 30;8:11435. doi: 10.1038/s41598-018-29294-w (PMC6065322; doi:10.1038/s41598-018-29294-w)
Supplement: Supplementary file 1 — SUPPLEMENTARY INFORMATION [file 41598_2018_29294_MOESM1_ESM.docx]

***Supporting content***

***Giant peak of the Inverse Faraday effect in the band gap of magnetophotonic microcavity***

**Mikhail A. Kozhaev,^1,2^ Alexander I. Chernov,^1,2^ Daria A. Sylgacheva,^1,3^ Alexander N. Shaposhnikov,^4^ Anatoly R. Prokopov,^4^ Vladimir N. Berzhansky,^4^ Anatoly K. Zvezdin,^2,5^ Vladimir I. Belotelov,^1,3,*^**

^1^ Russian Quantum Center, 45, Skolkovskoye shosse, Moscow, 121353, Russia

^2^ Prokhorov General Physics Institute RAS, 38 Vavilov Street, Moscow 119991, Russia

^3^ Faculty of Physics, Lomonosov Moscow State University, Leninskie Gory, Moscow 119991, Russia

^4^ Vernadsky Crimean Federal University, 4 Vernadskogo Prospekt, Simferopol, 295007, Russia

^5^ P.N. Lebedev Physical Institute of the Russian Academy of Sciences, 53 Leninskiy Prospekt, Moscow, 119991, Russia

Correspondence to [belotelov@physics.msu.ru](mailto:belotelov@physics.msu.ru)

**Derivation of Eq.(1)**

The magnetization dynamics of the magnetization M caused by the inverse Faraday effective magnetic field $\mathbf{H}_{\boldsymbol{IFE}}$ is governed by the Landau-Lifshitz-Gilbert equation. In the spherical coordinate system with *z*-axis along the normal to the film and *x*-axis along the in-plane external magnetic field *H* it is written by:

$\frac{\partial\theta}{\partial t}+\alpha_{G}\sin\theta\frac{\partial\varphi}{\partial t}=-\frac{\gamma}{M}\frac{\partial U}{\partial\varphi}\frac{1}{\sin\theta},$ (S1)

$-\alpha_{G}\frac{\partial\theta}{\partial t}+\sin\theta\frac{\partial\varphi}{\partial t}=\frac{\gamma}{M}\frac{\partial U}{\partial\theta},$ (S2)

where $\theta$ is the polar angle of magnetization, $\varphi$ is the azimuth angle of magnetization, $\gamma$ is the gyromagnetic ratio, $\alpha_{G}$ is the Gilbert damping constant and *U* is the free energy density of the magnetic film. In the case of the predominant growth anisotropy with respect to the crystalline one the free energy density *U* of the magnetic film is written by:

$U=-\left( 2\pi M^{2}-K_{U} \right)\sin^{2} \theta-HM\sin\theta\cos\varphi-\mathbf{M}\mathbf{H}_{IFE}(t)$. (S3)

The first term in Eq. (S3) describes the magnetic anisotropy caused by the planar shape of the magnetic sample and its growth. The magnetic anisotropy can be characterized by the effective anisotropy field $H_{a}=4\pi M-{2K}_{U}/M$. If the in-plane magnetic field *H* exceeds $H_{a}$ then in the absence of $\mathbf{H}_{IFE}$ the magnetization lies in-plane, which gives the equilibrium state:

$\theta_{0}=\pi/2$ and $\varphi_{0}=0$. (S4)

Passing to $\theta_{1}=\frac{\pi}{2}-\theta,$ linearizing Eqs. (S1) and (S2) and taking into account (S3) leads to the following set of equations:

$-\frac{\partial\theta_{1}}{\partial t}+\alpha_{G}\frac{\partial\varphi}{\partial t}=-\omega_{0}\varphi+\gamma H_{IFEy}\left( t \right)-\gamma H_{IFEx}\left( t \right)\varphi,$ (S4)

$\alpha_{G}\frac{\partial\theta_{1}}{\partial t}+\frac{\partial\varphi}{\partial t}=-\omega_{a}\theta_{1}-\omega_{0}\theta_{1}+\gamma H_{IFEz}\left( t \right)-\gamma H_{IFEx}\left( t \right)\theta_{1},$ (S5)

where $\omega_{a}=\gamma H_{a}$ and $\omega_{0}=\gamma H$. It follows from these equations that input of $H_{IFEx}$ in the excitation of the magnetization is negligibly small with respect to the input of the other $H_{IFE}$ components.

The problem of solving a set of non-homogeneous differential Eqs. (S4) and (S5) is equivalent to the problem of solving a set of homogeneous equation with initial conditions. Integration of Eqs. (S4) and (S5) over the duration of the pump pulse $\Delta t$ ($\Delta t\to0$) gives the following initial conditions:

$\theta_{1}\left( 0+ \right)\mathbf{=}\frac{-\gamma H_{IFEy}\Delta t+\gamma\alpha_{G}H_{IFEz}\Delta t}{1+\alpha^{2}},$ (S6)

$\varphi\left( 0+ \right)\mathbf{=}\frac{\gamma H_{IFEz}\Delta t+{\gamma\alpha}_{G}H_{IFEy}\Delta t}{1+\alpha^{2}},$ (S7)

where $H_{IFEy,z}$ are amplitudes of the IFE field pulses: $H_{IFEy,z}\left( t \right)=H_{IFEy,z}\Delta t\delta(t)$. The corresponding homogenous equation for $\theta_{1}$ is found from Eqs. (S4) and (S5) in the form:

$\frac{\partial^{2}\theta_{1}}{\partial t^{2}}+\frac{2}{\tau}\frac{\partial\theta_{1}}{\partial t}+\omega_{r}^{2}\theta_{1}=0,$ (S8)

where $\omega_{r}^{2}=\omega_{0}(\omega_{0}+\omega_{a})$, $\tau=2/\left[ \alpha_{G}(2\omega_{0}+\omega_{a}) \right]$. Its solution has the following form:

${\theta_{1}=\theta_{m}e}^{-\frac{t}{\tau}}\sin(\omega_{r}t+\beta)$*.* Taking into account the initial conditions (S6) and (S7) and Eq. (S4) one gets for $\alpha\ll1$ a set of algebraic equations for $\theta_{m}$ and $\beta$:

$\theta_{m}\frac{\omega_{r}}{\omega_{0}}\cos\left( \beta+\beta_{1} \right)=-\gamma\Delta tH_{IFEz}$, (S9)

$\theta_{m}\sin\beta=\gamma\Delta tH_{IFEy}$. (S10)

Solving these two simultaneous equations gives:

$\theta_{m}=\gamma\Delta t\sqrt{{{(H}_{IFEz})}^{2} \frac{\omega_{0}^{2}}{\omega_{r}^{2}}+(H_{IFEy})^{2}}$. (S10)

In the case of our experimental conditions (*H* = 890 Oe) $\frac{\omega_{r}}{\omega_{0}}\frac{H_{IFEy}}{H_{IFEz}}\sim0.2$. It allows to simplify Eq. (S10) to

$\theta_{m}=\gamma\left( 1+H_{a}/H \right)^{-1/2}H_{IFE}\Delta t$, (1)

where we denoted $H_{IFEz}$ by $H_{IFE}$.

**Calculation of the electromagnetic field distribution inside the magnetophotonic microcavity**

To calculate the distribution of $\mathbf{E}\mathbf{(}z\mathbf{)}$ inside the MPMC and its transmission and Faraday angle spectra we used the transfer matrix method^35^.

Light propagation inside multilayered structure can be fully described on the basis of the $4\times4$ matrix formalism. Let us consider 1D photonic structure consisting of $M$ magnetic and nonmagnetic layers and chose coordinate system with Z-axis perpendicular to the layers plane (Fig. S1).

*x*

*z*

0

Figure S1. One-dimensional multilayer structure.

In the $4\times4$ matrix formalism *proper modes* play an important role. A proper mode of light retains its polarization state during propagation through a material in a given direction. The proper modes of light in the magnetic media are circular polarized modes with the clockwise and anti-clockwise electric field rotation. Electric field inside $\text{ν}-th$ layer of the multilayer structure can be presented as a superposition of four proper modes:

,

where $\text{ε}_{\nu,P,d}$-is the modal amplitude; $n_{x,\text{ν}}$, $n_{y,\text{ν}}$, and $n_{z,\text{ν},P,d}$ are components of the normalized wave vector ; $k_{0}=\text{ω}/c$; $\vec{e}_{\nu,P,d}$ is the modal vector polarization. The first summation is taken on the two type of the proper mode polarizations: $r$ - right (clockwise) and $l$ - left (anti-clockwise) circular polarized waves. The second summation takes into account proper modes traveling along Z-axis ($d=+$) and in the opposite direction ($d=-$). A similar expression can be written for the magnetic field of the radiation in the $\text{ν}-th$ layer:

,

where , , $\text{η}_{0}=\sqrt{\text{μ}_{0}/\text{ε}_{0}}$, $n_{\text{ν},P,d}$ is the length of the normalized wave vector. The values of $\vec{e}_{\nu,P,d}$ and $n_{\text{z,ν},P,d}$ are determined as a solution of the wave equation obtained from the Maxwell equations:

.

Transfer matrix formalism operates with three main matrixes. The first one is $\mathbf{A}_{\text{ν}}$ - a column vector of four modal amplitudes $\text{ε}_{\nu,P,d}$:

,

where the subscript ^T^ denotes the transposed column vector. Other matrix is $\mathbf{D}_{\text{ν}}$ - dynamic matrix which relates modal amplitudes and tangential components of the electric and magnetic fields:

$\mathbf{D}_{\text{ν}}\mathbf{A}_{\text{ν}}=\left[\begin{matrix} E_{x,\text{ν}} & \text{η}_{0}H_{y,\text{ν}} & E_{y,\text{ν}} & \text{η}_{0}H_{x,\text{ν}} \end{matrix}\right]^{T}$.

The matrix $\mathbf{D}_{\text{ν}}$ is given by

.

The propagation of the modal amplitudes $\text{ε}_{\nu,P,d}$ in the layer is described by the propagation matrix $\mathbf{P}_{\text{ν}}$:

$\mathbf{A}_{\text{ν}}(z_{\text{ν}}+\text{δ})=\mathbf{P}_{\text{ν}}\mathbf{A}_{\text{ν}}(z_{\text{ν}+1}-\text{δ})$, $\text{δ}\to0$, where $z_{\text{ν}}$ and $z_{\text{ν}+1}$ are the boundaries of the $\text{ν-th}$ layer. The matrix $\mathbf{P}_{\text{ν}}$ writes

$\mathbf{P}_{\text{ν}}=\left[\begin{matrix} \exp\left[ik_{0}n_{z,\text{ν},r,+}a_{\text{ν}}\right] & 0 & 0 & 0 \\ 0 & \exp\left[ik_{0}n_{z,\text{ν},r,-}a_{\text{ν}}\right] & 0 & 0 \\ 0 & 0 & \exp\left[ik_{0}n_{z,\text{ν},l,+}a_{\text{ν}}\right] & 0 \\ 0 & 0 & 0 & \exp\left[ik_{0}n_{z,\text{ν},l,-}a_{\text{ν}}\right] \end{matrix}\right]$.

At our calculation it looks natural to deal with circular polarized proper modes, so the modal vector polarizations and their normalized wave vectors are given by

$\vec{e}_{\nu,r,\pm}=\left(\begin{matrix} 1 \\ \pm i \end{matrix}\right)$; $\vec{e}_{\nu,l,\pm}=\left(\begin{matrix} 1 \\ \mp i \end{matrix}\right)$;

$n_{\text{ν,}r,\pm}=\sqrt{\text{ε-g}}$; $n_{\text{ν,}l\text{,}\pm}=\sqrt{\text{ε}+\text{g}}$,

where $g$ is the gyration vector absolute value and $\text{ε}$ is the diagonal part of the ferromagnetic dielectric tensor $\hat{\text{ε}}=\left(\begin{matrix} \text{ε} & 0 & 0 \\ 0 & \text{ε} & 0 \\ 0 & 0 & \text{ε} \end{matrix}\right)+ig\left(\begin{matrix} 0 & -m_{z} & m_{y} \\ m_{z} & 0 & -m_{x} \\ -m_{y} & m_{x} & 0 \end{matrix}\right)$ (we neglect here the second order MO effects).

Before the multilayer structure (in the $0-\text{th}$ layer) and after the multilayer structure ($\left(M+1\right)-\text{th}$ layer) . Among six modal amplitudes in these expressions two amplitudes are known, namely $\text{ε}_{0,r,+}$ and $\text{ε}_{0,l,+}$ because they represent incident radiation. The other four amplitudes are to be found from the transfer matrix formalism.

In the absence of free charges and currents the tangential components of the electric field $\vec{E}(\vec{r}$ and magnetic field $\vec{H}(\vec{r}$ are continuous at the interfaces. The continuous tangential components of electric and magnetic fields are at the $\left(\text{ν}+1\right)-\text{th}$ interface (i.e. between the $\text{ν}-\text{th}$ and $\text{(ν}+\text{1)}-\text{th}$ layers) can be written in a matrix form

$\mathbf{D}_{\text{ν}}\mathbf{P}_{\text{ν}}\mathbf{A}_{\text{ν}}=\mathbf{D}_{\text{ν}+1}\mathbf{A}_{\text{ν}+1}$.

The recursive application of boundary conditions gives relation between the vector of modal amplitudes for the $0-\text{th}$ (for $z=0-\text{δ}$) and $\text{ν}-\text{th}$ layers (for $z=z_{\text{ν}+1}-\text{δ}$)

$\mathbf{A}_{\text{ν}}=\mathbf{S}_{\text{ν}}\mathbf{A}_{0}$,

where the matrix $\mathbf{S}_{\text{ν}}$ has the form

$\mathbf{S}_{\text{ν}}=\mathbf{P}_{\text{ν}}\left[\mathbf{D}_{\text{ν}}\right]^{-1}\mathbf{D}_{\text{ν}-1}\ldots\mathbf{D}_{2}\mathbf{P}_{2}\left[\mathbf{D}_{2}\right]^{-1}\mathbf{D}_{1}\mathbf{P}_{1}\left[\mathbf{D}_{1}\right]^{-1}\mathbf{D}_{0}$.

The relation between the column vectors of modal amplitudes before (in the $0-\text{th}$ layer) and after (in the $\left(M+1\right)-\text{th}$ layer) the multilayer structure can consequently be written as

$\mathbf{A}_{M+1}=\mathbf{S}_{M+1}\mathbf{A}_{0}$. (1)

Knowing the matrix $\mathbf{S}_{M+1}$, the reflection and transmission coefficients, and MO effects can be calculated.

To find reflection $R$ and transmission $T$ coefficients one needs to solve a set of four linear equations obtained from (1). Unknown values in this set are $\text{ε}_{0,r,-}$, $\text{ε}_{0,l,-}$, $\text{ε}_{M+1,r,+}$, $\text{ε}_{M+1,l,+}$. When modal amplitudes have been found it is straightforward to get

,

.

The Faraday rotation angle $\Phi$ can be written as

$\Phi=\text{tan}^{\text{-1}}\left(\frac{2\mathrm{Re}\text{χ}}{\text{1-}\left|\text{χ}\right|^{2}}\right)$,

where . The similar expression is valid for the Kerr rotation angle, but instead the reflected amplitudes should be used.
